# The Impact of Enterprise Management Elements on College Students’ Entrepreneurial Behavior by Complex Adaptive System Theory

**DOI:** 10.3389/fpsyg.2021.769481

**Published:** 2022-03-08

**Authors:** Yueyuan Cheng, Junlong Zhang, Yang Liu

**Affiliations:** ^1^English Department, Zunyi Medical University Zhuhai Campus, Zhuhai, China; ^2^Guangdong Guangzi International Engineering Investment Consultants Co., Ltd., Guangzhou, China; ^3^Institute of Education, University College London, London, United Kingdom

**Keywords:** complex adaptive system, entrepreneurial behavior, positive psychological intervention, emotion management, diversified management

## Abstract

At present, with the continuous rise in public consumption level, the pressure on college students’ entrepreneurship or employment is increasingly severe. Under the concept of positive psychological intervention, the present work aims to alleviate the entrepreneurial pressure of college students and improve college students’ entrepreneurial education through the analysis of enterprise management elements. A 3-month intervention experiment, including the pre-test, preventive curriculum intervention, post-test, and delayed test, is conducted on a control group and an experimental group, to investigate entrepreneurial intention, emotional management ability, and ability to deal with entrepreneurial pressure of college students. In addition, based on a complex adaptive system (CAS), the enterprise management elements are analyzed, and a three-layer network model is constructed. Meanwhile, new diversified elements of enterprise management are defined to discuss the effectiveness and psychological impact of diversified management, proving that psychological security plays an intermediary role in the cross-layer relationship chain in the three-layer CAS network. The experimental results indicate that on the whole, the positive psychological intervention reduces the pressure of students in the experimental group, significantly ameliorates depression and anxiety, and promotes the positive personality in all directions. Besides, in the delayed test after 3 months, the experimental group can maintain a relatively better state than the control group. By exploring the role effectiveness and characteristics of diversified management, this experiment confirms that the improvement of psychological security under positive psychological intervention has a positive impact on the effectiveness of diversified management. The present work discusses the hierarchical construction in enterprise management and puts forward reasonable suggestions and theoretical development for the influence of the entrepreneurial practice of college students.

## Introduction

As the social economy advances, the education of innovation and entrepreneurship has gradually attracted a proliferation of attention. However, it is accompanied by the even more severe employment and entrepreneurship pressure on college students, resulting in the negative emotions of college students, such as anxiety, tension, and confusion, which have a considerable impact on college students’ calm life (Li and Ashkanasy, 2019). If such negative emotions cannot be regulated in time, college students will inevitably have psychological barriers or psychological problems, in the long run, ultimately leading to many psychological problems such as a decline in endurance (Taylor, 2019). In this case, they cannot successfully achieve the role transition from students to employees, or even undergo physical and mental ailments (Han, 2019). For students, psychological distress can hinder their physical and mental development, causing a great loss to universities and society. The emergence of positive psychology advocating positive orientation is a crucial revolution to psychology (Song, 2019). It pays attention to positive psychological quality instead of injury and defect in psychology that previous research focuses on. Positive psychology takes maximum advantage of the intervention effects of positive prevention to effectively stifle potential psychological problems and negative signals in the cradle (Veloso et al., 2019). Based on the concept of positive prevention under positive psychological intervention, a questionnaire survey and an experimental intervention are performed here to study entrepreneurial intention, emotion management ability, and the ability to cope with entrepreneurial pressure (Esteves et al., 2021).

Furthermore, the new multiple leadership structure of enterprise management is showing strong adaptability in a complex environment (Wallerstein et al., 2019). This structure enables enterprises to take initiative to respond to different circumstances and changes fast (Rosa et al., 2019). The theory of multiple leadership originated in the 1920s (Pidun et al., 2019). It suggests that it is difficult for a single manager to lead all members to achieve team goals, and group members and managers need to lead each other to guide each element of the structure to participate in the team management process (Huang et al., 2019). This diversified, decentralized, and networked management structure has emerged, accompanied by the diversified role of all members of the enterprise in the process of corporate management, and the group capability has begun to replace the personal heroic leadership (Bressan and Weissensteiner, 2021). Numerous educational institutions, management teams, consulting services enterprises, and high-tech research and development teams have tried to popularize such a management structure in recent years. Besides, such enterprise management elements are very innovative for the current entrepreneurship education and entrepreneurial behavior of college students (Beuselinck et al., 2019).

The present work is structured as follows. Section “Introduction” elaborates the diversified enterprise management and complex adaptive system (CAS) theory and constructs a three-layer CAS model of diversified management. Section “Literature Review” designs the questionnaire of college students’ entrepreneurial pressure and a 3-month positive psychological intervention experiment for the experimental group and the control group as the research object. Section “Diversified Enterprise Management Based on Complex Adaptive System and Entrepreneurial Behavior Under Positive Psychological Intervention” analyzes the impact of positive psychological intervention on college students’ entrepreneurial behavior and the influence of different psychological safety on CAS enterprise management elements. The principal innovation of the present work is employing psychological intervention to study the entrepreneurship and employment of college students and implementing a questionnaire survey and psychological intervention course experiment to investigate the entrepreneurial pressure of college students. Besides, the impact of different psychological safety factors on the role of enterprise management elements of CAS is analyzed. The research content and results can provide corresponding psychological guidance and suggestions for entrepreneurship and employment of college students.

## Literature Review

Lyndon et al. (2020) studied the impact of cognitive trust on shared leadership and the mediating effect of team learning on the relationship between shared leadership and team creativity. The authors performed a questionnaire survey to collect the data of 44 teams at two different time points and conducted semi-structured interviews with 22 of them. Through analysis, they found that cognitive trust had a positive impact on shared leadership, multiple leadership played a positive role in team innovation, and team creativity could be enhanced by promoting common leadership in the organization. Hao and Long (2020) conducted a questionnaire survey on the paired data of 69 departments and 262 employees to explore the impact and mechanism of shared leadership on employees’ initiative to change behavior. Through the cross-level analysis, they found that shared leadership had a positive impact on employees’ proactive change behavior; meanwhile, the role definition of proactive change behavior and harmonious work passion would, respectively, mediate the relationship between shared leadership and employees’ proactive change behavior. Moreover, the quality of leader-member transfer would, respectively, adjust the relationship between shared leadership and employees’ role width, self-efficacy, and harmonious work passion. The higher the quality of the leader-member transfer, the more significant the positive effect of shared leadership on employees’ role width, self-efficacy, and harmonious work passion. Besides, the quality of leader-member transfer would adjust the indirect effect of shared leadership on employees’ proactive change behavior through role width and self-efficacy. The higher the quality of the leader-member transfer, the more significant this indirect effect. Stephens et al. (2021) reported that entrepreneurship education, network, and incubation space have provided students with direct information to help them make entrepreneurial decisions, as well as situational, collaborative, and ubiquitous indirect information clues. They also noted the multifaceted and dynamic nature of students’ entrepreneurial process and discussed the potential impact of these factors on students’ decision-making process. Stephens analyzed the significance of the survey from the perspective of researchers and educators, pointing out the challenges faced by student entrepreneurs in the educational and entrepreneurial environment far away from society. Burnette et al. (2019) randomly selected undergraduate students in the introductory course of entrepreneurship (*N* = 238) were divided into a growth mentality intervention group and a knowledge-based attention matching control group. After the intervention experiment, the authors found that compared with the control group, students in the growth mentality intervention group showed higher entrepreneurial self-efficacy and task persistence in their main classroom projects. Besides, the intervention also indirectly improved academic and professional interest through entrepreneurial self-efficacy. Kusumojanto et al. (2021) adopted a quantitative method with a cross-sectional survey model to understand how entrepreneurship education, family education, environment, and entrepreneurial attitude explain vocational students’ entrepreneurial intention. The scholars took vocational students in Malang, Indonesia as a sample for analysis by using the model structure and iterative theory of the structural equation modeling. They confirmed that students’ environment could explain their entrepreneurial intention and attitude. Duval et al. (2021) proposed a curriculum model of science and technology entrepreneurship education to enable academic researchers to play a more active and informed role in the commercialization of their discovery. This curriculum could address the problems faced in the process of Entrepreneurship, namely technology readiness and timing, intellectual property pathway decisions, engagement with the entrepreneurial ecosystem, and personal career choices. Chen et al. (2021) systematically analyzed the research on hybrid and online entrepreneurship learning and teaching. They found that the current research status and achievements of scholars on the educational technology used in online entrepreneurship education and hybrid entrepreneurship education could be categorized into social media, serious games, and large-scale open online courses. They selected five examples from the three educational technologies and evaluated them using the scoring table to compare these technologies. Through experiments, they proved that the large-scale online open curriculum could furnish a platform and high-quality learning resources. Besides, considering the advantages and challenges of applying each technology to entrepreneurship education, teachers and learners needed to successfully select the most appropriate technology combination to achieve the objectives of the entrepreneurship curriculum. Ndofirepi (2020) studied whether psychological characteristics (achievement needs, risk-taking tendency, and internal control points) mediated the predictive relationship between the perceived effect of entrepreneurship education and entrepreneurial intention, and they investigated 308 vocational education students in Zimbabwe. The results showed that there was a significant positive correlation between the impact of entrepreneurial education variables and achievement needs, risk-taking tendency, internal control sources, and entrepreneurial goal intention. In addition, achievement needs, risk-taking tendencies, and internal control points statistically and significantly explained the differences in entrepreneurial intention. However, among the three psychological characteristics, only achievement needed partially mediate the relationship between entrepreneurship education effect and entrepreneurship goal intention.

Here, a three-layer network model is established based on CAS theory to analyze the diversified management structure of enterprises. Moreover, the effectiveness results are combined with experiments to explore the influence of the positive psychological intervention on college students’ entrepreneurial behavior.

## Diversified Enterprise Management Based on Complex Adaptive System and Entrepreneurial Behavior Under Positive Psychological Intervention

### Diversified Enterprise Management and Complex Adaptive System Theory

After the 1990s, management teams of enterprises began to develop a flat team model, and diversified enterprise management has also developed rapidly. Consequently, management concepts with diverse characteristics are generated, such as shared leadership, distributed leadership, collective leadership, and collaborative leadership. Although these terms are different, in essence, they have a common feature, i.e., diversity (Santoro et al., 2019). Management power in an enterprise is not exclusive to a particular individual, but the product of the interaction between team members (Chen, 2019). The above management concept is defined as the management network created by a collective interaction between all individuals through formal or informal relationships, emphasizing the development and change of management power in enterprises from individual heroism to group participation mode. Therefore, three characteristics of the diversified enterprise management can be summed as the participation of multiple roles in the group, the multiple relationship interaction between formal and informal, and the directionality of social network generated by interaction.

The theory of CAS emphasizes that under the interaction between the subject and other subjects or the environment, the subject realizes learning and growth by changing its structure, to achieve the perfection of the system (Yan et al., 2019). The definition indicates that the subject can grow continuously because of its adaptive characteristics, and the adaptability of the subject is the essence of CAS. From a micro perspective, CASs are not adaptive subjects that can follow environmental changes, and they have certain integral characteristics. Since there are non-linear relationships between individuals and individuals or between individuals and the environment in the system, it is necessary to study this theory from a holistic perspective. Adaptability is the most prominent and critical feature of the CAS, meaning that the subject can adjust the state of the subject itself according to the influence of the subject, individual, or environment (Liu et al., 2019), to adapt to the influence and achieve the goal. Existing CASs have four recognized characteristics, namely aggregation, non-linearity, fluidity, and diversity. Among them, aggregation means that individuals at lower levels will form higher-level individuals through aggregation behavior. Non-linearity shows that the impact of individuals on the system is complex and non-linear. The information flow, material flow, or energy flow existing between the individual and the individual or between the individual and the environment reflects its characteristic of fluidity. Moreover, after being affected, subjects tend to differ with different characteristics, showing obvious gaps and diversity (Wu W. et al., 2020). Different from the previous passive and mechanical system theory, CAS can learn and accumulate continuously with the help of initiative and adaptability to optimize its structure for the adaptation to changes. Such a system provides innovative ideas for recognizing, understanding, and controlling management, so it is often used in the management field. CAS theory holds that any organization is a complicated adaptive system formed by the interaction between subjects. Therefore, the diversified enterprise management network can also be seen as a CAS composed of continuous interaction and influence between managers and subordinates (Yuan and Wu, 2020). The interaction among the individual, binary relation, and group constitutes diversified management. Therefore, these three elements are taken as analytical units to construct a three-layer CAS network.

### Definition of Enterprise Management Elements and Modeling of the Complex Adaptive System Network

**FIGURE 1:**
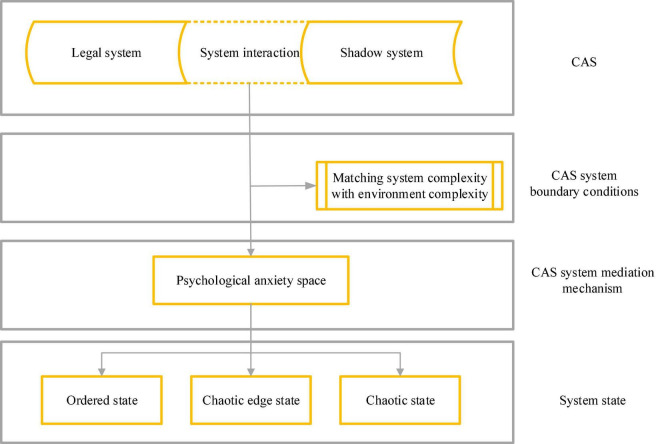
CAS theoretical research framework.

In the CAS of diversified management, formal interaction between groups to maintain the stability and unity of the business network constitutes a legal system, while informal interaction that promotes changes in the system forms a shadow system. The continuous non-linear interaction between the two systems makes CAS produce three different states of order, disorder, and chaotic edge, resulting in various phenomena, such as insignificance, low efficiency, high efficiency, and high creativity of diversified management efficiency (Chen et al., 2020). In the mental space theory of CAS, it is the legal system that follows the formal regulation of the organizational system and maintains its order. This orderly system can effectively inhibit the psychological anxiety of groups of the organization. The shadow system is a spontaneous informal interactive group among members within the organization, which is uncertain and unstable and may exacerbate the psychological anxiety of members (Liu et al., 2021a). The continuous non-linear interaction between the two systems leads to different psychological anxiety states of the internal groups of the organization. System complexity and environmental complexity in CAS indicate that when the system is as complex as the environment, it can cope with the complicated changes in the environment. Figure 1 illustrates the theoretical research framework constructed based on the CAS theory.

**FIGURE 2:**
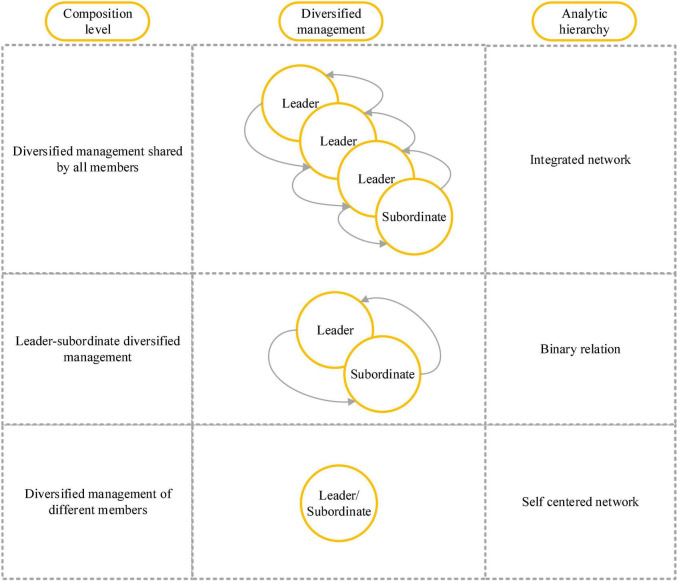
The diversified management model of three-layer CAS network.

Diversified management is a management network formed by the interaction of formal and informal activities of organizational groups at multiple levels, including individuals, binary relations, and groups (Liu and Chen, 2021). Diversified management networks at different levels separately constitute CASs. Figure 2 reveals the diversified management model of the three-layer CAS network under the foundation of the definition of diversified management.

In Figure 2, the three layers of the model represent the multi-role participation of group members as the essence of diversified management. This work discusses the different management roles of diversified management in managers or subordinates, in the relationship between the manager and the subordinate, and different management roles in groups. Diversified management at the individual level lays the foundation of diversified management, which can reflect the management efficiency of each member in the organizational group. Each manager or subordinate plays a unique management and leadership role in the organization group, and the difference is only the specific effects of different individuals. Diversified management in the binary relation occurs in the interaction between managers and subordinates through informal social networks, showing the diversified management of the relationship between the two parties. The diversified management at the group level is the management ability and effectiveness of all members, occurring between managers, between managers and subordinates, and between the subordinates, which is the process of interaction between formal networks and informal networks.

Diversified management is defined by synthesizing the characteristics of diversified management. At the individual level, its unique diversified management efficiency is defined through the formal or informal self-centered networks formed by the relationship between each member and other members of the group (Liu et al., 2021b). At the binary relation level, the instrumental and emotional formal or informal interactions between managers and subordinates are used to define the diversified management efficiency. At the group level, the effectiveness of diversification management is defined through formal and informal networks formed by member groups (Wu J. et al., 2020).

Four participating elements of diversified management are defined at the individual level based on CAS (Sun et al., 2020), namely, vagrants, experts, participants, and innovators. Among them, the vagrant represents that when an individual is in a weak chaotic CAS state of the formal system and shadow system, the individual is dissociated from the organization and rarely participates in the activities of enterprise management. Experts indicate that an individual in an orderly state of CAS becomes an expert in the organizational activities of enterprise management with the help of management efficiency. Participants demonstrate that an individual in an informal and orderly CAS state will extensively get involved in management activities. The innovator indicates that when an individual is in a strong chaotic edge CAS state of the legal system and shadow system, he or she will lead group members of activities (Zheng and Ke, 2020).

The four interaction elements of diversified management at the binary relation level based on CAS are defined as disordered interaction, expert-supported interaction, emotional developmental interaction of subordinates, and group creative interaction. The disordered interaction means that when the binary relation is in the weak chaotic CAS state of the legal system and shadow system, there are very few instrumental interaction behaviors, and there are few informal social interaction behaviors and relationships between managers and subordinates. The expert-supported interaction indicates that when the binary relation is in a formal and orderly CAS state, the instrumental interaction happens to complete the work, and the relationship between managers and subordinates increases. The emotional developmental interaction of subordinates means that in the state of informal ordered CAS, the subordinate will strive to establish an intimate relationship with the manager and produce emotional interactions, to increase informal social relationships. In this way, the subordinate can better convey the ideas and problems found from the perspective of the subordinate. The group creative interaction indicates that when the interactive relationship is in a strong chaotic edge CAS state of the legal system and shadow system, instrumental and emotional interaction will occur simultaneously in quantity. Then, informal social relations between managers and subordinates will continue to increase, as well as expert-supported interaction and emotional interaction (Vazquez and Lvarez-Delgado, 2020).

The four network elements of diversified management at the group level based on CAS are the free network, expert network, engagement network, and group network. The free network means that there are rare formal and informal social interactions in the chaotic CAS state, so the management network presents disorder. The expert network indicates that in an orderly formal state, influential managers will control the activities of most members of the group. The engagement network demonstrates that in the informal and order state, the managers who are good at informal social interaction will show a positive desire for communication in the management network and express their innovative ideas. The group network indicates that in the chaotic edge CAS state, managers and subordinates become participants showing innovation and group strength in competition and cooperation (Ferhatoglu and Yapici, 2020).

According to the definition of elements in diversified management based on CAS, the aggregation means and forms of entrepreneurial talents in the entrepreneurial behavior of college students can be summarized as low-level subjects gathering to form high-level subjects (Wu and Song, 2019). Besides, the macro factors of entrepreneurship education and entrepreneurial environment can be concluded as the influence of the external environment on subjects. The implementation model of entrepreneurship education is the interaction of information flow in CAS, and the individuality of teachers and students is the diversity of subjects.

### Design of College Students’ Entrepreneurial Pressure Questionnaire and Positive Psychological Intervention

#### Research Object

The relevant psychological curriculum content is formulated to improve the coping ability and emotional management ability of college students and release their potential abilities in the face of entrepreneurial pressure. It is hoped to guide students to a good mental state through positive psychological guidance (Feng and Chen, 2020), and reduce psychological contradictions under entrepreneurial pressure by optimizing and enhancing individual psychological quality and stress coping ability of students. A total of 312 students from two undergraduate colleges in G city in the grade of 2019 (junior year) are selected as research objects, of which 151 students consist of the experimental group, and 161 constitute the control group. The research objects contain 137 males and 175 females, all aged between 19 and 21. The following assumptions are made before the questionnaire survey.

Hypothesis 1: the entrepreneurship and employment pressures of college students have nothing to do with psychological factors.
Hypothesis 2: psychological intervention hurts college students’ entrepreneurship and employment.
Hypothesis 3: psychological counseling course plays a positive role in college students’ psychological construction.

#### Questionnaire Design

Table 1 illustrates the entrepreneurial pressure questionnaire as the basis for the three tests.

**TABLE 1:** Entrepreneurial pressure questionnaire.

Questionnaire type	Main content	Scoring system
Psychological stress Scale for college students	Self-pressure; social pressure.	5-Point rating. The higher the score, the more pressure.
Coping strategies questionnaire (CSQ)	Solving problems; remorse; seeking help; fantasy; escape; rationalization.	Six indicators. The higher the score, the higher the frequency of use.
Self-rating anxiety scale (SAS)	Frequency of anxiety symptoms.	4-Level rating. Scores more than 50 indicate anxiety symptoms.
Self-rating depression Scale (SDS)	Frequency of depressive symptoms.	4-Level rating. Scores more than 53 indicate depression symptoms.
Sixteen personality factor questionnaire 16 (PF)	Personality factor test.	Different personality factors are obtained according to the score formula.

#### Curriculum Design of Positive Psychological Intervention

The psychological quality theory curriculum is a critical channel of college students’ psychological quality optimization. Here, the curriculum primarily explains psychological knowledge and skills and introduces the basic methods of psychological quality adjustment and training. This curriculum aims to inspire students to actively learn to understand and deal with the pressure that they may encounter in entrepreneurship and to cultivate their environmental adaptability and psychological adjustment ability (Secundo et al., 2021). There are a total of 12 theoretical courses, with the contents involving the interpretation of the meaning of mental health, confirmation of self-consciousness, shaping good personality, emotional management, cognitive pressure, interpersonal communication, interview psychology, environmental adaptation, crisis response, frustration education, cherishing life, and love psychology.

The psychological counseling practice curriculum is complementary to the theoretical knowledge curriculum. The real-world exercises and group activities in this curriculum enable students to feel the mutual connection and strength between groups, thus improving their emotional management ability and resilience facing external environmental pressure (Deng et al., 2021), and releasing anxiety and depression caused by pressure through group relations. A total of 12 practical courses are designed, including mental health awareness establishment, self-awareness activities, personality shaping activities, emotional venting, stress relief, interpersonal communication practice, simulated interviews, entrepreneurial scenario simulation, adaptability training, adversity behavior response, frustration scenario simulation, and multiple-choice scenario simulation (Liu and Qi, 2021).

### Experimental Procedure

**FIGURE 3:**
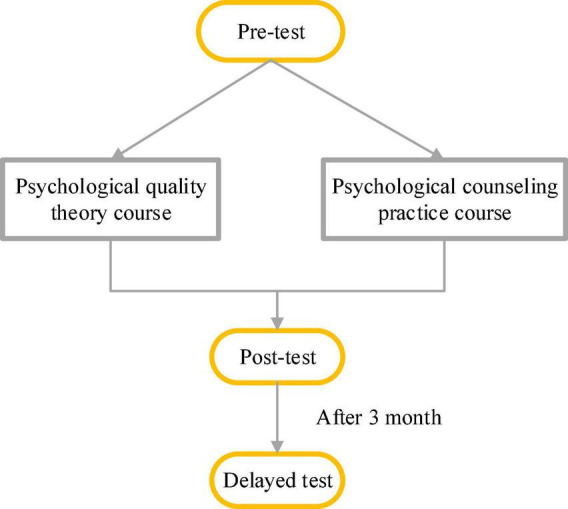
Experimental procedure.

Figure 3 represents the experimental procedure.

There are 151 respondents in the experimental group and 161 in the control group.

(1) Pre-test: The experimental group is tested by the Psychological Pressure Scale, CSQ, SAS, SDS, and 16PF for college students, and the results are recorded. The control group is tested by the same scales, and the results are recorded.
(2) Preventive curriculum intervention.
The students in the experimental group are given a 3-month positive psychological training course twice a week, containing a theoretical explanation and a group activity practice, a total of 24 times. The course is taught by a professional psychological teacher. Besides, the researcher contacts the class counselor to ensure that all students in the experimental group participated in the training course. The students in the control group have no course training.
(3) Post-test: After the course, all experimental group students’ basic situation is tested. Three months later, the basic situation of all students of the control group that do not participate in the course training is tested.
(4) Delayed test: Three months after the post-test, a delayed test is conducted on the students in the experimental group to check whether the training contents such as psychological quality optimization can alleviate the employment pressure and improve the emotional management ability of college students. Besides, the intervention effects of the pre-test and the post-test are compared. The delayed test after 3 months can determine whether the intervention effect is maintained, whether the entrepreneurial pressure has been alleviated, and what impact it has on students’ entrepreneurial behavior. The students in the control group also undergo the delayed test.

## Experimental Results and Discussion

### Experimental Results and Analysis of Effects of Positive Psychological Intervention on the Entrepreneurial Behavior of College Students

**FIGURE 4:**
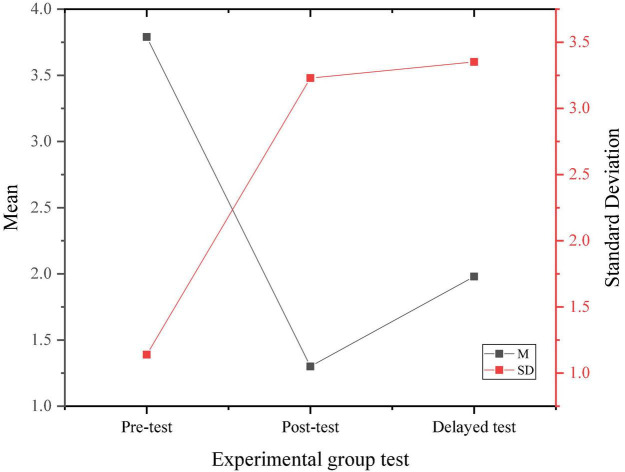
Results of three psychological stress tests of the experimental group.

**FIGURE 5:**
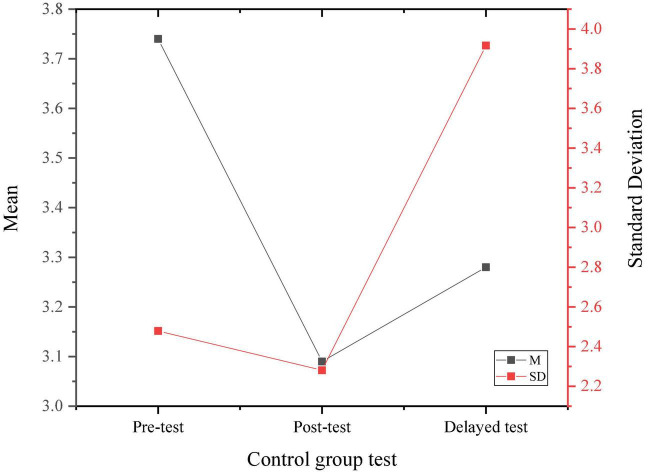
Results of three psychological stress tests of the control group.

Figures 4, 5 display the three psychological stress tests of the experimental group and the control group according to the implementation of the experiment and the survey results of the experimental group and the control group.

**FIGURE 6:**
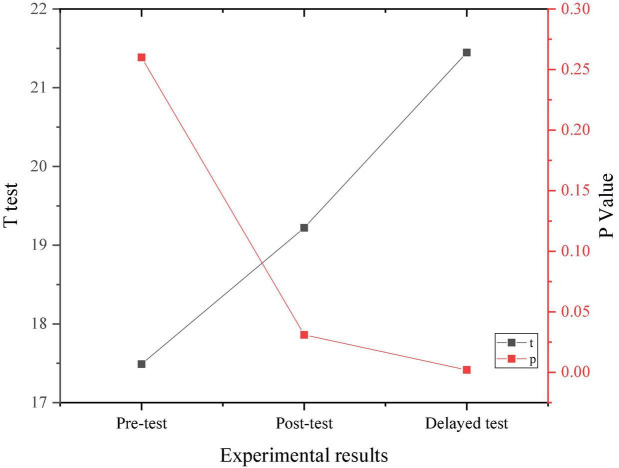
Comparison between the experimental results of two groups.

Figure 6 signifies the comparison of the experimental results of the two groups.

Through the data results from Figure 6, the randomly selected students show a medium level of psychological stress as a whole, and there is no significant difference in the performance of the pre-test, indicating the homogeneity of the test members. After a 4-month positive psychological intervention curriculum, the psychological pressure on research objects shows an overall downward trend, proving that the positive psychological intervention curriculum has a positive effect on the entrepreneurial pressure on college students.

**FIGURE 7:**
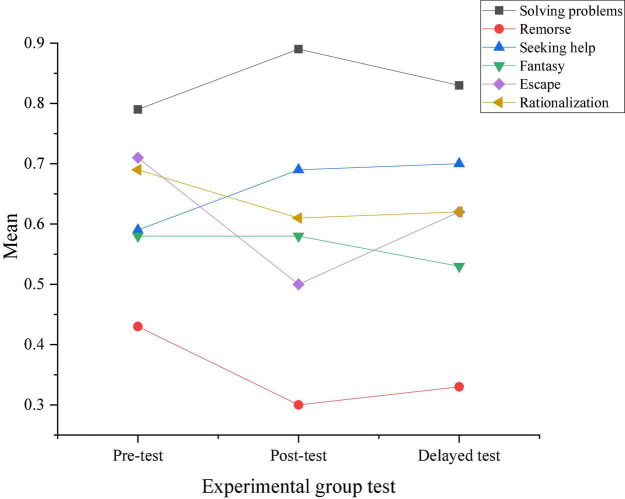
Results of three tests of coping ability in the experimental group.

**FIGURE 8:**
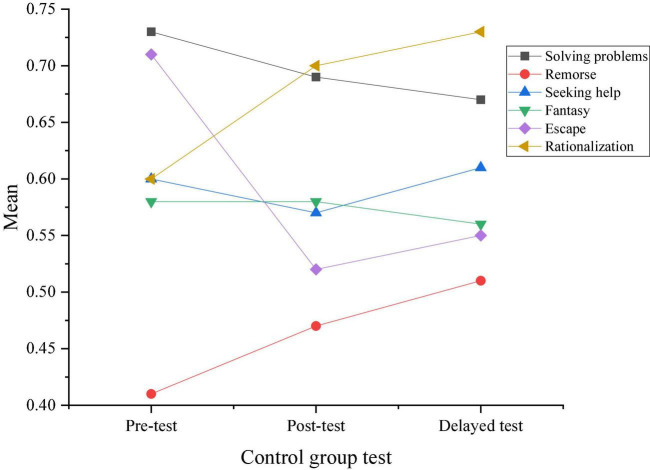
Results of three tests of coping ability in the control group.

Figures 7, 8 provide the results of three tests by the CSQ questionnaire for the coping ability in the two groups. On this basis, the changes in response choices of the two groups before and after the intervention experiments are obtained.

**FIGURE 9:**
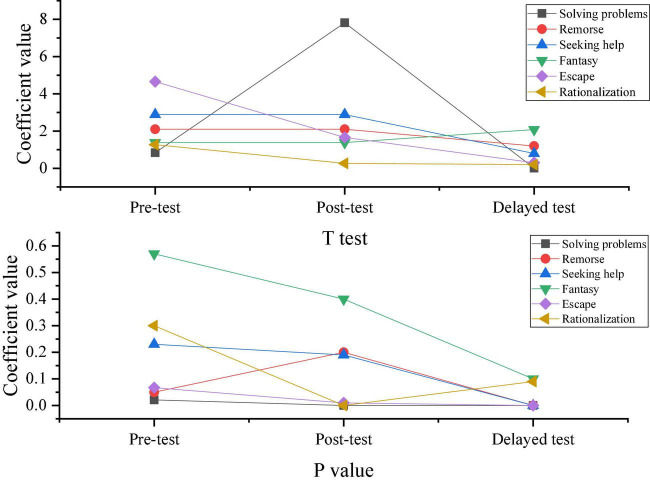
Comparison of response choices between the experimental group and the control group.

Figure 9 describes the comparison of response choices between the experimental group and the control group according to the results shown in Figures 7, 8.

According to the results shown in Figure 9, students who did not take the positive psychological intervention curriculum tend to choose negative or neutral coping styles, and this trend is also maintained in the delay test after 3 months. Moreover, students who have received positive psychological intervention generally take positive responses, and the difference between the two groups is gradually significant in the post-test.

**FIGURE 10:**
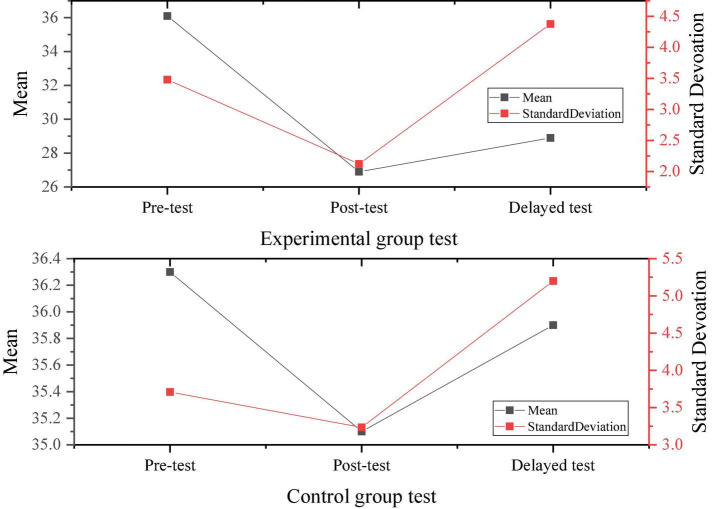
Comparison of anxiety of the experimental group and the control group.

Figure 10 shows the comparison of anxiety degree of research objects combined with the survey results of the SAS questionnaire and three tests.

**FIGURE 11:**
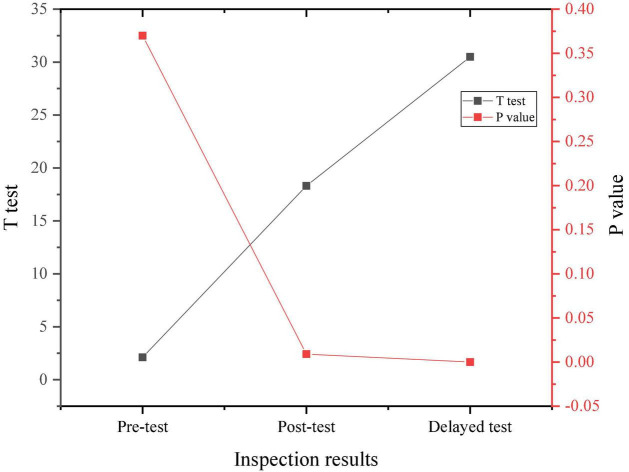
Statistical results of anxiety of the experimental group and the control group.

Figure 11 provides the statistical test results of anxiety of two groups according to the results in Figure 10.

From Figure 11, there is no significant difference in the anxiety level between the experimental group and the control group before the intervention experiment. After the positive psychological intervention, the anxiety level of the students in the experimental group shows a downward trend and remains lower than that of the students in the control group in the delay test after 3 months.

**FIGURE 12:**
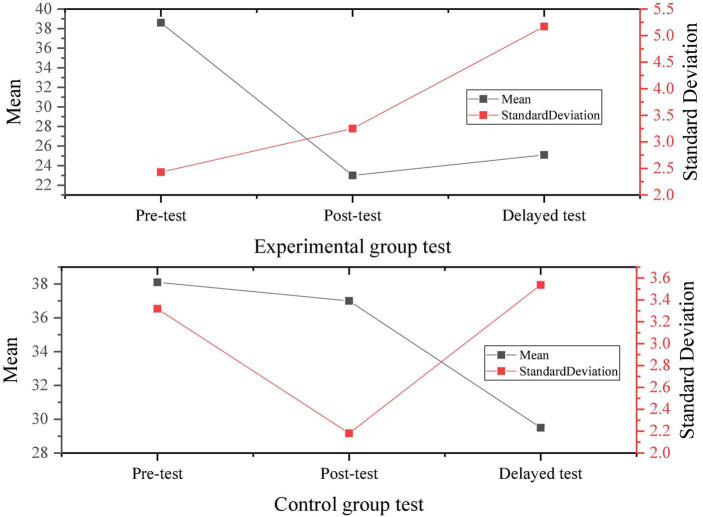
Comparison of the depression degree of the experimental group and the control group.

**FIGURE 13:**
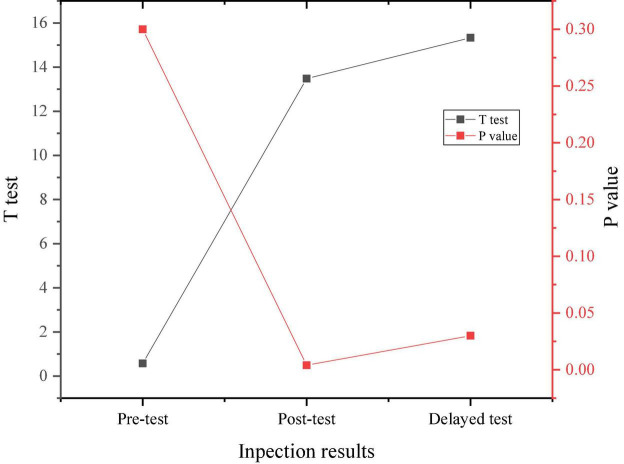
Statistical results of the depression degree of the experimental group and the control group.

According to the survey results of the SDS questionnaire and data calculation, the results of the depression degree of the two groups of samples are compared, as shown in Figures 12, 13.

Through the comparison of the above results, before the psychological intervention experiment, there is no significant difference in the depression degree of the two groups. After the experiment, the depression degree of the students in the control group is significantly higher than that of the students in the experimental group, and there is a significant difference in statistics. Therefore, it can be inferred that psychological anxiety and depression of students will gradually decrease by learning theoretical knowledge and skills of psychological quality and improving psychological quality. In the face of environmental changes in the entrepreneurial situation, positive psychological intervention can effectively regulate students’ tension and anxiety, while alleviating their depression and adjusting the psychological state and psychological conflict. Positive psychological intervention can not only create a stable and nice coping state for individuals, but also improve their antidepressant ability. The college students selected as sample groups in the test are homogeneous in terms of psychological stress, depression, and anxiety before the experiment, showing an intermediate level. After the positive psychological intervention experiment, the students in the experimental group are significantly improved in terms of psychological stress, depression, and anxiety, with the *t*-value of 19.214 and the *p*-value less than 0.05, indicating that the positive psychological intervention curriculum has a positive effect on the entrepreneurial pressure of college students. Therefore, Hypothesis 1 and Hypothesis 2 do not hold, and Hypothesis 3 holds. Scholars in related fields have also conducted some research work. For example, Liu and Qi (2021) conducted a comprehensive analysis of the relationship between physical exercise and positive mental health indicators such as positive emotions, happiness, happy quotient, positive psychological quality, and successful aging based on the perspective of positive psychology. However, due to the excessive concentration of research objects, the sampling is not scientific enough, the research paradigm lacks strict experimental design, and the research results are not universal. Moreover, the research on the relationship between physical exercise and happy quotient, social happiness, and positive psychological quality is relatively insufficient, and the conclusion of causality is lacking. In this work, the concept of positive psychology is utilized to conduct an intervention study on the quality of employment pressure response and emotional management ability of college students. The experiment sets up a control group and lasts for 6 months. The experimental results indicate that after the intervention of positive psychology courses, the overall psychological pressure of the experimental group students has been significantly reduced.

### Analysis of the Effects of Different Psychological Security on Complex Adaptive System Enterprise Management Elements

**FIGURE 14:**
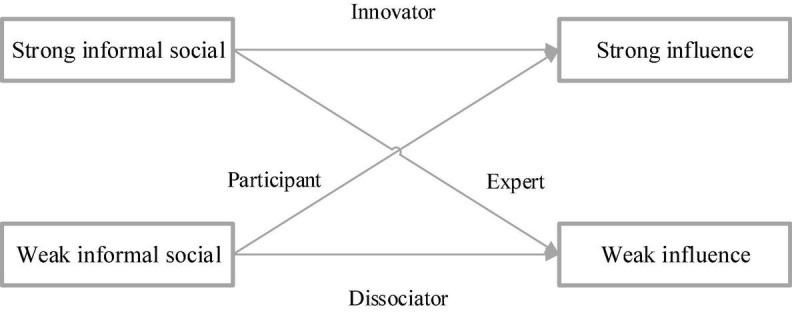
Relationship at the individual level.

**FIGURE 15:**
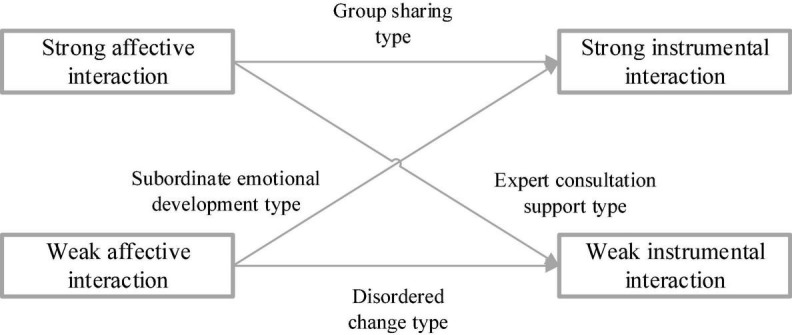
Relationship at the binary relation level.

**FIGURE 16:**
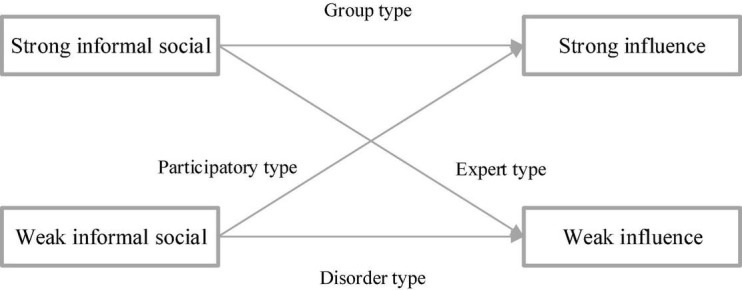
Relationship at the group level.

Figures 14–16 represent the influence relationship of management elements at each level based on the analysis of the three-layer CAS network.

**FIGURE 17:**
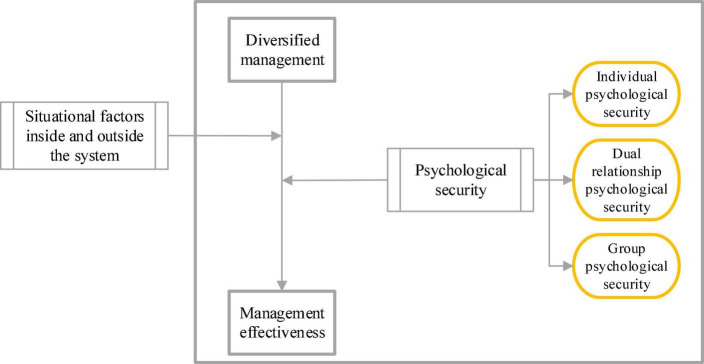
Impacts of psychological security.

From Figures 14–16, when the diversity of the concept, belief, personality, and psychological state of subordinates in the diversified management network increases, the interaction between the legal system and the shadow system of the diversified management network will become more complex. Consequently, the psychological anxiety of subordinates is affected, and the management efficiency of diversified management decreases. In addition, psychological security is taken as a function of CAS behavior, and the legal system is regarded as a shared state in CAS, which can maintain the *status quo* and inhibit the psychological anxiety brought about by innovation and facilitate the completion of tasks. The shadow system is a spontaneous state formed by the individual, so it is easier to innovate and destroy it in behaviors such as social activities and fantasy, resulting in psychological anxiety, stress, and conflict. Figure 17 denotes the mediating effect of psychological security.

When the diversity of subordinates increases, the individual’s self-management effectiveness, and informal social interaction will produce heterogeneous information in the interaction. Meanwhile, the two types of social interaction between managers and subordinates will become more diversified, and the overall management efficiency and informal social interactions will become more complex, thus transmitting more contradictions and conflicts. Diversified management at the individual, binary relation, and group levels has led to more contradictions and conflicts due to the increase in diversity of formal and informal interactions, thus triggering emotional conflicts, increasing anxiety, and reducing psychological security.

To sum up, the individual psychological security of vagrants in the CAS network is not significant, and meanwhile, they are too disorderly to show significant management efficiency. In contrast, experts have high individual psychological security, and they can show efficient management efficiency. Therefore, the dominant legal system can effectively suppress the psychological anxiety caused by the shadow system. The shadow system of participants tries to break the legal system and bring innovative results, so it is prone to bring psychological anxiety and low personal psychological security, resulting in low management efficiency. The legal system of innovators can restrain their psychological anxiety, while the shadow system can bring inspiration and psychological anxiety and pressure. The tension caused by such interaction will maintain moderate psychological anxiety and ensure that no emotional flooding behavior occurs to create space, showing the most significant management efficiency.

## Discussion

Section “Experimental Results and Analysis of Effects of Positive Psychological Intervention on the Entrepreneurial Behavior of College Students” analyzes the entrepreneurial pressure of college students through the positive psychological intervention experiment. Ge et al. (2020) proposed a method to find mental health problems by analyzing the data sets of students’ campus behavior, including Internet access logs, dormitory access records, and canteen consumption records. They established a classification model for distinguishing mental health problems between students and normal students via the collected data. The experimental results showed that the established classification model helped college students find their problems, thus easing the employment pressure such as entrepreneurship; however, it took a long time as a whole, which was not conducive to dealing with the pressure problems in time. Moreover, group psychological counseling can create a harmonious and united friendly atmosphere in which members can share experiences, form the belief of mutual assistance, and jointly promote the realization of group goals. In addition, team counseling also offers a platform for interpersonal communication and interaction, enabling members to express their views, realize the collision of ideas, and absorb new knowledge in the collision. In this harmonious atmosphere, it is easier for members to open their hearts and present their points. It is also easier to encourage and support each other and jointly resist setbacks and difficulties. In general, this way of career group counseling can effectively improve students’ self-efficacy of career decision-making and alleviate students’ overall psychological pressure to a certain extent, but it is difficult to verify the long-term effect of the intervention. The study reported here found that the overall psychological pressure of college students participating in psychological intervention courses shows a downward trend, indicating that positive psychological intervention courses play a positive role in college students’ entrepreneurial pressure. The reason is that the psychological intervention course dredges the pressure of subjects in the face of entrepreneurship and employment from the perspective of psychology. Therefore, subjects are more likely to choose a positive way and mentality to deal with this stress, and this effect still exists after 3 months. Meanwhile, the psychological intervention experiment also ameliorates the anxiety of college students. The reason is that active psychological intervention can effectively regulate students’ tension and anxiety in the face of the change of entrepreneurial environment, alleviate their depression, and manage their psychological state and psychological conflict. The research results in section “Analysis of the Effects of Different Psychological Security on Complex Adaptive System Enterprise Management Elements” prove that positive psychological intervention can regulate individual psychological safety, and psychological safety plays a positive intermediary role between the management efficiency and hierarchical elements of the CAS. This is also the uniqueness of the present work.

## Conclusion

In conclusion, based on the above positive psychological intervention experiment and CAS modeling analysis, positive psychological intervention can effectively alleviate the entrepreneurship pressure on college students and guide college students to actively respond to environmental changes and frustration crises encountered in the process of entrepreneurship. Besides, mastering psychological skills and quality improvement is conducive to reducing the anxiety and depression levels of college students in entrepreneurship activities. The positive psychological curriculum intervention has a positive effect on college students who are about to face the employment evaluation situation, whether in the way of coping with stress or shaping good personality traits. After the intervention of a positive psychological curriculum, some positive factors of students’ personality, such as gregariousness, intelligence, stability, courage, independence, and self-discipline, have been improved to a certain extent. To sum up, positive psychological courses have a moderating effect on alleviating the stress of college students, and anxiety and depression of students decrease with the mastery of psychological skills and the improvement of psychological quality after the intervention. Positive psychological intervention can effectively regulate the psychological problems of individual tension, anxiety, and irritability, and facilitate emotional management to help individuals achieve a strong and stable coping state.

Positive psychological intervention can also regulate individual psychological security that plays a positive mediating effect between management efficiency and hierarchical elements in the CAS network. In the entrepreneurial education of college students, it is feasible to strengthen the formal and informal interactions between managers and subordinates bidirectionally, to balance the contradiction between subordinates’ creative efficiency and psychological anxiety. Correspondingly, more chaotic edge management roles can be produced, which are conducive to the formation of creativity. A positive psychological intervention course verified by experiments is designed in this report for entrepreneurship education and psychological security of college students. Moreover, this work provides effective data and practical reference for the construction of psychological security of managers and subordinates in CAS diversified enterprise management. However, there are still some limitations in the present work. Follow-up research will strengthen the measurement and test of the effectiveness of external environmental networks to provide social indicators for different levels. In addition, in the future, it is necessary to study the relationship between personalized training and college students’ entrepreneurship, and comprehensively study individual differences.

## Data Availability Statement

The raw data supporting the conclusions of this article will be made available by the authors, without undue reservation.

## Ethics Statement

The studies involving human participants were reviewed and approved by the Zunyi Medical University Zhuhai Campus Ethics Committee. The patients/participants provided their written informed consent to participate in this study. Written informed consent was obtained from the individual(s) for the publication of any potentially identifiable images or data included in this article.

## Author Contributions

All authors listed have made a substantial, direct, and intellectual contribution to the work, and approved it for publication.

## Conflict of Interest

JZ was employed by Guangdong Guangzi International Engineering Investment Consultants Co., Ltd. The remaining authors declare that the research was conducted in the absence of any commercial or financial relationships that could be construed as a potential conflict of interest.

## Publisher’s Note

All claims expressed in this article are solely those of the authors and do not necessarily represent those of their affiliated organizations, or those of the publisher, the editors and the reviewers. Any product that may be evaluated in this article, or claim that may be made by its manufacturer, is not guaranteed or endorsed by the publisher.
